# New preclinical models for angioimmunoblastic T-cell lymphoma: filling the GAP

**DOI:** 10.1038/s41389-020-00259-x

**Published:** 2020-08-14

**Authors:** Rana Mhaidly, Adrien Krug, Philippe Gaulard, François Lemonnier, Jean-Ehrland Ricci, Els Verhoeyen

**Affiliations:** 1 Université Côte d’Azur, INSERM, C3M, 06204 Nice, France; 2 Université Paris-Est Créteil; Institut Mondor de Recherche Biomédicale, INSERMU955; Institut Mondor de Recherche Biomédicale, INSERMU955, Université Paris Est Créteil, Créteil, France; 3 Département de Pathologie, Hôpitaux Universitaires Henri Mondor, Assistance publique des Hôpitaux de Paris, Créteil, France; 4 Unité Hémopathies Lymphoïdes, Hôpitaux Universitaires Henri Mondor, Assistance Publique des Hôpitaux de Paris, Créteil, France; 5 CIRI, Université de Lyon, INSERM U1111, ENS de Lyon, Université Lyon1, CNRS, UMR 5308, 69007 Lyon, France; 6 Present Address: Institut Curie, Stress and Cancer Laboratory, Equipe Labellisée par la Ligue Nationale contre le Cancer, PSL Research University, 26, rue d’ULM, F-75248 Paris, France; 7 Present Address: Inserm, U830, 26, rue d’ULM, Paris, F-75005 France

**Keywords:** Cancer models, Non-hodgkin lymphoma, Targeted therapies

## Abstract

Mouse models are essential to study and comprehend normal and malignant hematopoiesis. The ideal preclinical model should mimic closely the human malignancy. This means that these mice should recapitulate the clinical behavior of the human diseases such as cancer and therapeutic responses with high reproducibility. In addition, the genetic mutational status, the cell phenotype, the microenvironment of the tumor and the time until tumor development occurs, should be mimicked in a preclinical model. This has been particularly challenging for human angioimmunoblastic lymphoma (AITL), one of the most prominent forms of peripheral T-cell lymphomas. A complex network of interactions between AITL tumor cells and the various cells of the tumor microenvironment has impeded the study of AITL pathogenesis in vitro. Very recently, new mouse models that recapitulate faithfully the major features of human AITL disease have been developed. Here, we provide a summary of the pathology, the transcriptional profile and genetic and immune-phenotypic features of human AITL. In addition, we give an overview of preclinical models that recapitulate more or less faithfully human AITL characteristics and pathology. These recently engineered mouse models were essential in the evaluation of novel therapeutic agents for possible treatment of AITL, a malignancy in urgent need of new treatment options.

## Angioimmunoblastic T-cell lymphoma: a challenging complex malignancy

### Clinical aspects

**Fig. 1:**
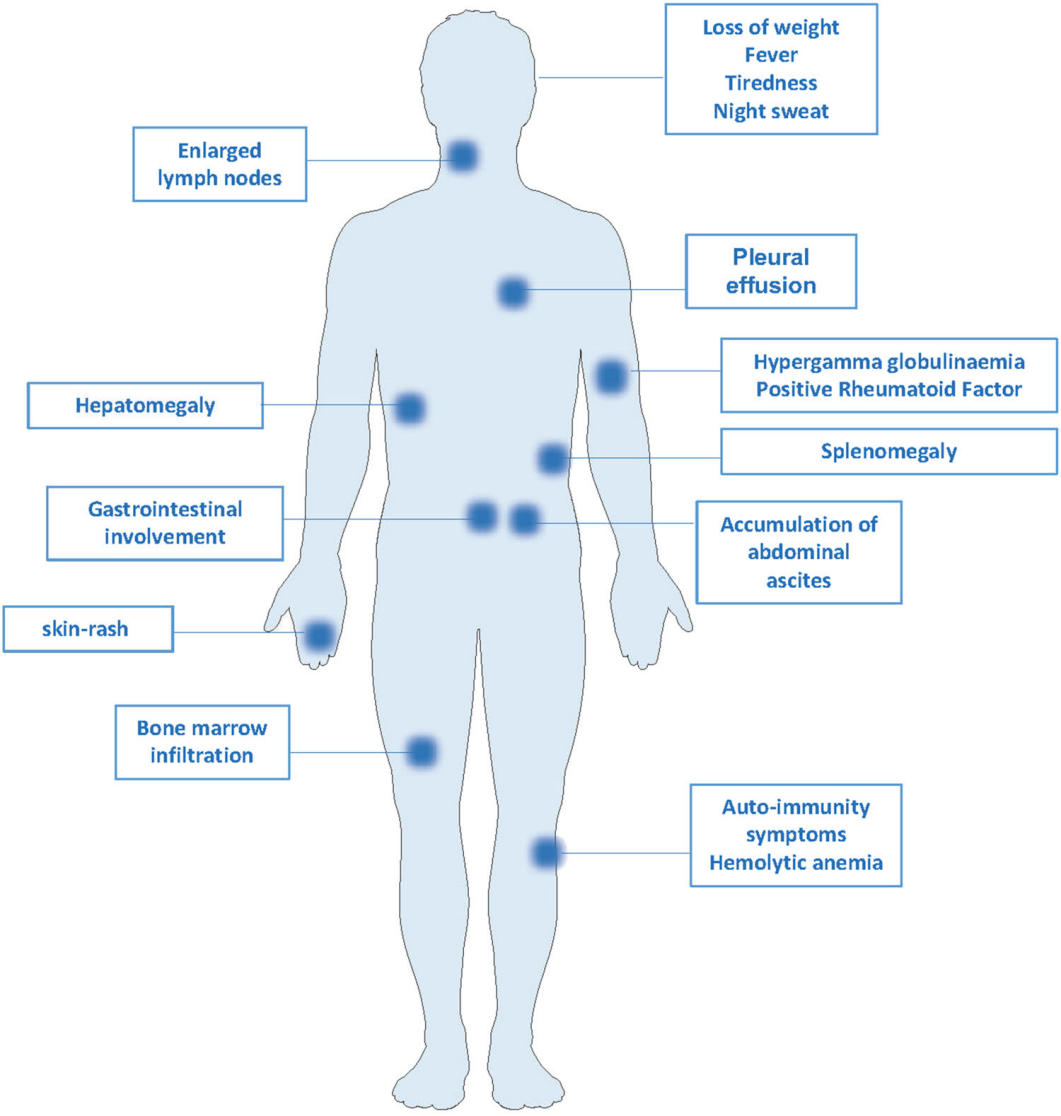
Clinical features of AITL patients. Different clinical manifestations of AITL in patients are indicated.

Angioimmunoblastic T-cell lymphoma (AITL) belongs since 2016 after revision of the world health organization (WHO) classification to the nodal T-cell lymphomas with follicular helper T-cell (Tfh) phenotype^1^. AITL is a rare, aggressive lymphoma with little treatment options. Overall survival is generally poor with a five-year median survival rate of 32%^2,3^. It represents only 1–2% of the non-Hodgkin’s lymphomas. However, AITL appears to be the most prevalent PTCL, representing up to 35% of the non-cutaneous peripheral T-cell lymphomas^2,4–6^. AITL pathology develops late in life with a median age at onset of 59–65 years and patients display a generalized lymphoadenopathy^7,8^. Seventy percent of the patients have bone marrow involvement and unfortunately early stage detection of AITL is very uncommon (10%)^9^. Disease symptoms resemble closely an infection inducing immunologic hyper-activation (fever, rash, loss of weight, hemolytic anemia^2,5^) (Fig. 1).

AITL has particular clinical and pathological features, associated with splenomegaly, hepatomegaly and enlarged lymph nodes showing effacement of normal architecture and appearance of endothelial venules^3^ (Fig. 1). Other typical clinical manifestation of AITL are cutaneous lesions also called skin rash in 20–50% of the patients and accumulation of abdominal ascites^5,10–13^. These patients often test positive for autoimmunity including detection of rheumatoid factor, anti-smooth muscle, and nuclear autoantibodies^14^. They also present a general increase in immunoglobulins called hypergammaglobulemia (30%)^7,12^.

### The pathogenesis and immuno-phenotype of the AITL tumors emphasizes the importance of the tumor microenvironment

**Fig. 2:**
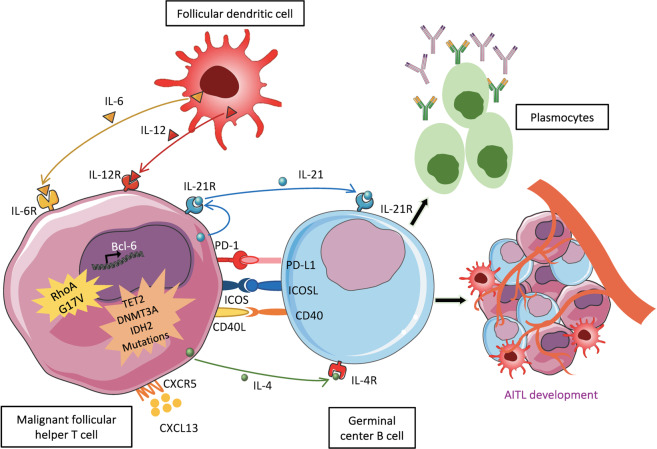
A schematic representation of the AITL lymphoma development. The cell of origin in AITL is considered to be a follicular helper T cell, which is in strong interaction with the tumor microenvironment including germinal center B cells and follicular dendritic cells.

The cell of origin for AITL lymphoma is the T follicular helper cell (Tfh), an effector T-cell subset^15–17^. Tfh cells play an important role in autoimmunity and this coincides with the autoimmune features and B-cell proliferation in AITL tissue. However, Tfh cells do not make up the majority of the AITL tumor tissue since massive infiltration of accessory cells occurs^3^, with up to 90% contribution by cells of the microenvironement^18^. Cytokines and chemokines released by the Tfh cells may indeed recruit immune cells into the tumor^15^. Immune cells like non-tumor reactive CD4 and CD8 T cells, B cells, eosinophils, macrophages, follicular dendritic cells (FDCs) invade the malignant tissue^3,19^ (Fig. 2).

Current understanding is that CD4^+^ Tfh cells in AITL are very similar to healthy Tfh cells. Both are characterized by a co-expression of markers: the chemokine receptor CXCR5, programmed cell death (PD1) and the inducible T-cell co-stimulator (ICOS) on their surface and high levels of intracellular nuclear transcription factor B-cell lymphoma 6 (BCL-6)^20–23^ (Fig. 2). These Tfh molecules are for the moment most frequently used and robust markers to confirm AITL diagnosis. One can add to that a high-level expression in the cytoplasm of the chemokine CXCL-13, which is secreted by healthy and AITL Tfh cells. The CXCL-13 chemokine binds to its receptor CXCR5, which promotes the recruitment of B cells and FDCs into AITL tissue^15^. The FDCs are often associated to venules and identified by CD21, CD23, or CD35 markers^24^.

Tfh cells also secrete IL-21 and IL-4, two cytokines contributing to B-cell proliferation in the germinal center^25^. Interestingly, CD10 a surface molecule expressed by both neoplastic and normal Tfh, delineates a subset of IL-4 producing Tfh cells^26^. Witalis et al.^27^ recently highlighted the importance of T-B-cell interaction in AITL-like murine tumors. Mast cells in this tissue produce VEGF, a cytokine inducing angiogenesis and recruitment of endothelial cells^28^ (Fig. 2). All these different cell types form the AITL tumor microenvironment (TME). The strong interaction between the Tfh tumor cells and the TME is intriguing since it seems that these cellular components are essential for the establishment and maintenance of the pathology.

### AITL presents T cell and sometimes B-cell clonality

**Fig. 3:**
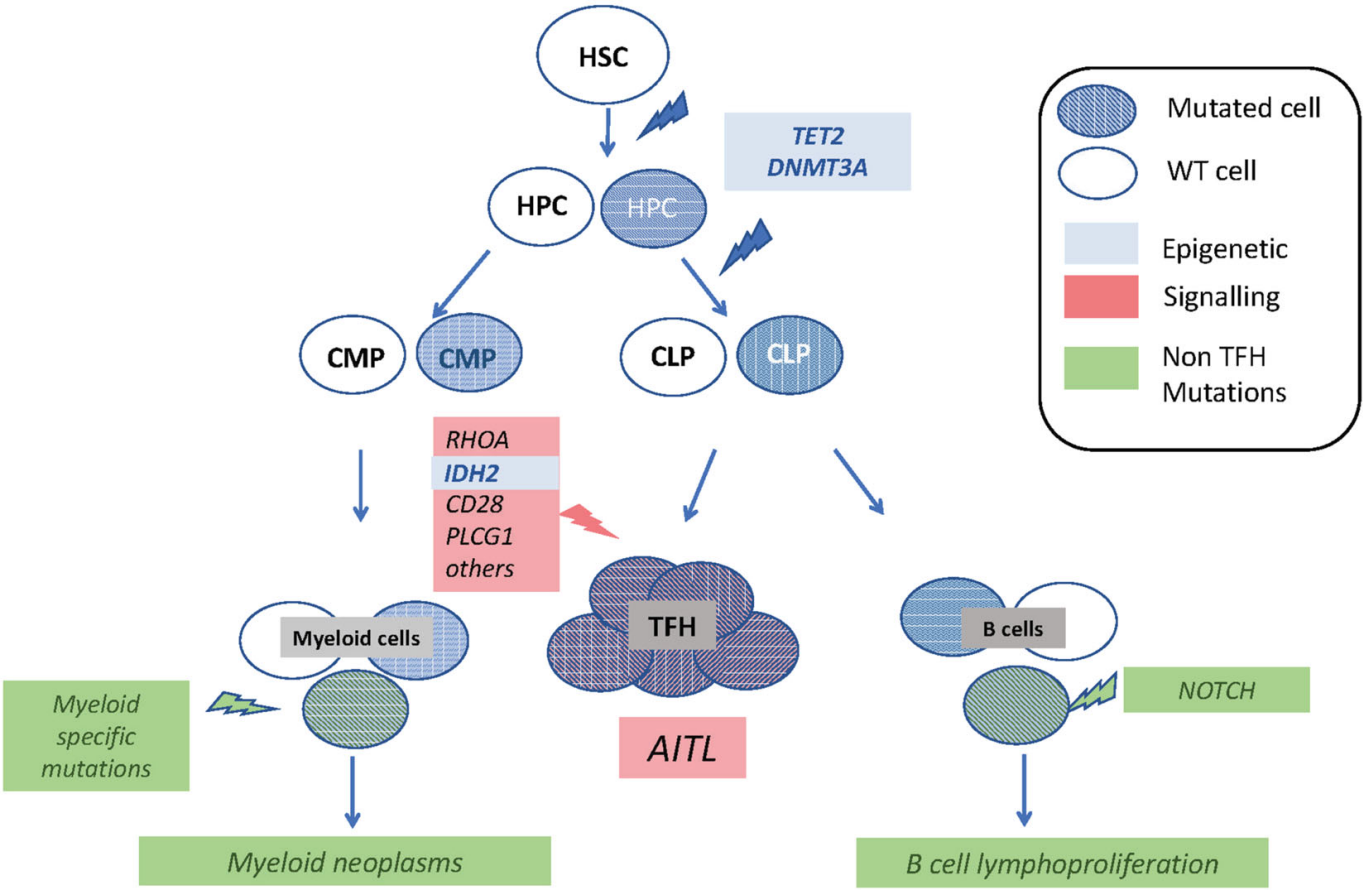
Mutational hierarchy in AITL development and its relationship with other cancers. *Tet2* and *DNMT3A* mutation are found in the hematopoietic stem cells (HSC) and hematopoietic progenitors (HPC), which are passed to the common myeloid progenitors (CMP) and myeloid lineages, which can accumulate other mutation and lead to myeloid malignancies such as CMML and AML. In parallel, *Tet2* and *DNMT3A* mutations are passed to common lymphoid progenitors (CML), which accumulate additionally specific mutations in genes important for T-cell function (e.g., RhoA, IDH2, CD28, PLCG1 and others) leading to AITL. In some cases, additional B-cell specific mutations such as *NOTCH1* mutations occur in addition to *Tet2* and *DNMT3A* mutations resulting in B-cell malignancies.

Early molecular studies using PCR techniques confirmed that for the vast majority (90%), T cells in AITL biopsies demonstrated a clonal rearrangement of the TCRγ chain. A monoclonal T-cell configuration was observed in over 75% of AITL patients^29–33^. Later on, Mao et al.^34^ demonstrated that AITL Tfh cells contain a clonal V segment of the TCRβ chain. Importantly, also the B-cell component of the tumor associated with the Tfh can show clonal rearrangements of the BCR^30^. This confirmed that B cells play a major role in AITL pathogenesis and explained why some AITL patients developed B-cell lymphoproliferative disorder and most frequently, diffuse large B-cell lymphoma (DLBCL)^35^. Importantly, Nguyen et al.^36^ demonstrated that clonality of T and B cells in AITL might be due to different mutation(s) in each of these compartments. They showed that *RHOA* and *IDH2* mutations were confined to the PD1^+^ T cells while a *NOTCH1* mutation was exclusively detected in the B cells of an AITL patient (see the “AITL mutational hierarchy” section and Fig. 3).

In addition, a strong correlation between Epstein bar infected B cells and AITL pathogenesis has been established. EBV-positive B cells have been detected in 66–86% of patients with AITL^29,37^. These tumor infiltrating B cells sometimes show monoclonal IgG rearrangements and might subsequently progress to an EBV-positive B-cell lymphoma^35,38^. This is of importance for therapeutic interventions in these patients since EBV (re-)activation can occur^39,40^. It remains controversial whether EBV status has an impact on the survival of AITL patients^41,42^. In addition, it has not been proven nor excluded that EBV^+^ status is a causal event in AITL^14,43^.

### Frequent genetic aberrations in AITL

Recent genetic studies identified in AITL recurrent mutations in rash homology family member A (*RHOA*) (50–70%)^44–46^ and genes encoding for epigenetic modifiers, tet methyl cytosine dioxygenase 2 (*TET2*; 47–84%)^45,47^, DNA methyltransferase 3 alpha (*DNMT3A*; 20–30%)^44,45,48^, and isocitrate dehydrogenase 2 (*IDH2*; 20–45%)^45,49^ as well as components of the T-cell receptor (TCR)-signaling pathway^50,51^ (Fig. 2).

#### The GTPase *RHOA* is mutated in the majority of AITL patients

Importantly, AITL is characterized by a recurrent, almost exclusive, *RHOA*^G17V^ substitution present in up to 70% of the patients^44–46^. This *RHOA*^G17V^ mutation is also commonly observed in other T-cell lymphomas with Tfh phenotype and are seldom detected in other cancers^48^. Therefore, it serves as a genetic indicator for Tfh lymphoma detection. RHOA belong to a family of small GTPases responsible for connecting cell surface receptors to intracellular effector proteins involved in migration, signaling, proliferation, and survival. Thus, not surprisingly, it is important for multiple T-cell functions and modulation of T-cell receptor signaling^52–55^. RHOA in its active state will bind guanine triphosphate (GTP) and in its inactive state guanine diphosphate (GDP). The switch between these two states relies on guanine exchange factors^56^. The mutant *RHOA*^G17V^ shows impaired GTP loading, which leads to altered RHOA signaling^44–46^. More recently, a novel mutation in *RHOA* (K18N) has been identified in 3% of AITL patients. In contrast to the *RHOA*^G17V^ mutation, *RHOA*^K18N^ also affects the binding site to GTP but in this case by increasing the affinity of RHOA for its substrate and stabilizing in this way its active form^51^.

Many studies support that RHOA has an essential role during T-cell development^52,57^. However, the mechanisms and functions of RHOA action in T cells are not yet completely revealed. The existence though, in high frequency of this point mutation in *RHOA* suggests that it might play a role in AITL oncogenesis. Only very recently the role of the specific *RHOA*^G17V^ mutation in T cell and AITL tumor development was investigated using knock-in mice for this mutation, a mouse model discussed below^54,58^ (see the “Inactivation of Tet2 combined with overexpression of RhoA G17V” section). Using these models, a clear relationship between this mutation *RHOA*^G17V^ and Tfh differentiation in CD4 T cells was elucidated, which confirms a link with AITL pathology.

#### *TET2* loss-of function mutations in AITL patients

Analysis of the mutational landscape of AITL showed *TET2* loss-of function mutations in up to 80% of AITL patients^45,47^. TET2 converts methylation cytosine into hydroxylmethyl cytosine (5hmC), formylcytosine and carboxylcytosine. These modified cytosines can then ultimately be excised and replaced by unmodified cytosines to achieve active demethylation^59–61^. Furthermore, 5hmC was reported to be criticial in the activation of enhancers and control of gene expression^62^. In AITL patients, many nonsense and frameshift mutations are found throughout the entire *TET2* sequence, but missense mutations are mostly restricted to the C-terminal catalytic domain^45,47^. This indicates that these *TET2* mutations are loss-of-function mutations. However, how TET2 mutations drive lymphomagenesis is poorly understood.

The current dogma attributed the role of TET2 in hematopoiesis and hematological malignances to its DNA demethylase activity, while TET2 non-enzymatic functions remained unstudied. Ito et al.^63^ demonstrated using transgenic mice models that TET2 demethylase activity is critical for myelopoiesis, while its non-enzymatic functions play a role in hematopoietic stem cell maintenance, lymphopoiesis and tumor suppression. This suggests that catalytic and non-catalytic functions of TET2 contribute distinctively to myeloid and lymphoid malignancies^63^. These mutations are also observed in healthy elderly people with clonal hematopoiesis. In a mouse model with single *TET2* knock-out, increased hematopoietic stem cell renewal was detected and differentiation was biased toward the myeloid lineage but did not necessarily develop blood malignancies^64–67^. A more recent *Tet2*^−/−^ mouse model, which is described in detail below (see the “Inactivating Tet2: the Tet2^−/−^ mouse model” section), developed in aged mice a T-cell lymphoma with typical Tfh features^66^.

Overall, the identification of frequent *TET2* mutations in AITL and other Tfh-related peripheral T-cell lymphomas (PTCLs) extends the importance of epigenetic alterations in T-cell lymphomagenesis.

Remarkably, most of the AITL patients carrying the *RHOA*^G17V^ mutation also have *TET2* mutations^45,46^ indicating a cooperation between these two mutations in disrupting normal CD4 T-cell phenotype and function in this aggressive lymphoma. This clearly suggests that impaired RHOA function in combination with TET2 loss of function, most probably preceding the RHOA mutation, leads to AITL pathogenesis. This was very recently confirmed by three independently developed genetic animal models that are detailed below^55,58,68^. These transgenic mouse models reproduce multiple features of AITL and might be considered as the first valid preclinical AITL mouse models.

#### *DMT3A* mutations in AITL

DNMT3A is an enzyme that catalyzes the transfer of a methyl group on cytosine residues in the CpG dinucleotide leading to repression of target gene expression. In AITL patients, inactivating mutations in *DNMT3A* were identified in 20–30% of the cases^69,70^. A hotspot mutation (p.R882H) in *DNMT3A* accounts for 15% of the *DNMT3A* mutations in AITL. This mutation was shown to have decreased methyltransferase activity and act as a dominant negative inhibitor of wild-type DNMT3A^71,72^.

Interestingly, in some patients double mutations in *TET2* and *DNMT3A* are involved in malignant transformation although they have opposite epigenetic effects^73^.

#### *IDH2* mutations in AITL

*IDH2* mutations are found in AITL, myeloid tumors, and gliomas. Gliomas are even classified according to their IDH status (IDH-mutant or wild-type gliomas)^74,75^. Interesting glioma patients carrying *IDH* mutations show a favorable prognosis compared to their *wt* counterparts, possibly because the IDH mutation effects provides a wider therapeutic window^76–78^. In AITL, *IDH* mutations are restricted to *IDH2* arginine (R) 172^45,49^. Moreover, the *IDH2*^R172K^ mutation is exclusively found in AITL and not in other T-cell lymphomas emphasizing a specific role of the mutated protein in this pathology^45,79,80^. Moreover, *IDH2* mutations are restricted to the neoplastic T cells not to others cells of the tumor microenvironment^81^ The non-mutated IDH enzymes convert isocitric acid to alpha-ketoglutarate (α-KG). The latter is an intermediate metabolite of the TCA cycle. Mutations in isocitrate dehydrogenases, *IDH1* and *IDH2*, contribute to malignant progression by producing the aberrant oncometabolite, D2-hydroxyglutarate (D2-HG)^49,82^. D2-HG inhibits the α-KG dependent enzymatic activity of dioxygenases, to which belongs the TET family proteins and histone demethylases^83^. This affects directly the production of 5-hydroxymethylcytosine and leads to the augmentation of cytosine methylation of certain genomic regions leading to a repression of target genes. However, though *IDH2*^R172^ mutations affect TET2 function and most *IDH2* mutated AITL also harbor *TET2* mutations, *TET2* and *IDH2* mutations are mutually exclusive in acute myeloid leukemia^83^. This suggests that *IDH2*^R172^ might take part in installing the lymphoma through its proper action not implicating TET2^80^. In this context D2-HG can inhibit more than 60 dioxygenases dependent on α-KG, which are involved in multiple cellular functions including epigenetic regulation, HIF1α regulation and collagen maturation^84^. Importantly, deregulation of these cellular functions as a consequence of *IDH2* mutations might lead to oncogenesis.

#### Mutations in TCR-signaling genes in AITL

First, the *RHOA*^*G17V*^ mutant, can bind to vav guanine nucleotide exchange factor 1 (VAV1), resulting in VAV1 phosphorylation and NFAT signaling activation, inducing T-cell proliferation. This indicates that the *RHOA*^*G17V*^ mutation could be a major player in T-cell signaling activation^57^. In addition, many other components of the TCR-signaling pathways such as phospholipase Cγ1 (14%), CD28 (9–11%), Src family tyrosine kinase (FYN) (3–4%), and VAV1 (50%) are mutated in AITL.

Multiple mutations in *CD28* have been revealed in 10% of AITL patients. It is well known that CD28 is a co-stimulation receptor expressed at the T-cell surface. Two specific *CD28* mutations have been identified (D124 and T195), which induce a prolonged activation of T cells. The mutated CD28 receptor represents an extremely high affinity for its ligand leading to a constitutive activation of a cascade of signaling pathways implicated in T-cell proliferation and cytokine production^50,85^. Moreover, a fusion gene *CD28-CTLA-4* encoding for the CD28 cytoplasmic domain and the CTLA-4 extra cellular domain, as also a CD28-ICOS fusion gene were recently identified in AITL^86^. Unexpectedly, expressed on T cells, this fusion gene can convert the normal inhibitory signals induced by CTLA-4 stimulation in a signal that activates T cells^87^.

FYN is a tyrosine kinase that also plays an essential role in T-cell activation. In AITL lymphoma, multiple mutations in FYN have been detected, which invalidate the inhibitory function of the FYN domain SH2, resulting in a constitutive activation of the tyrosine kinase and T-cell activation^44^.

Many more mutations that are implicated in the stabilization of stimulatory signals in T cells have been described in AITL such as mutations in *PLCG1, CARD11, CTNNB1*, as also proteins involved in the PI3K or MAPK pathways. All of these mutations favor T-cell proliferation and survival. One interesting point though is that *CARD11* mutations seem to be at the origin of constitutive NF-κB signaling and might assist in tumor outgrow^51^.

#### AITL mutational hierarchy

*TET2* and *DNMT3A* mutations were first described in myeloid neoplasms, such as acute myeloid leukemia (AML), myelodysplastic syndrome or chronic myelomonocytic leukemia (CMML), with CMML carrying the highest frequency of *TET2* mutations^88^. More recently, they were also described in clonal hematopoiesis of elderly people without detectable hematological disease, a condition called clonal hematopoiesis of indeterminate potential (CHIP)^89,90^. In AITL, *TET2* and *DNMT3A* mutations can be detected not only in neoplastic T cells, but also in B cells^36^ and CD34^+^ stem and progenitor cells^67^, suggesting that these events occur at an early stage during AITL lymphomagenesis (Fig. 3). In contrast, *RHOA* and *IDH2* mutations are restricted to neoplastic PD1-positive cells, suggesting a multi-step mutational hierarchy in AITL oncogenesis^36,81^ (Fig. 3). Interestingly, co-existence of myeloid disorders such as CMML and AITL have been described, at a higher frequency than expected^40,91,92^. Both malignancies share common ancestral mutations in *TET2* and/or *DNMT3A*, while disease specific mutations result in the emergence of the AITL (RHOA, TCR signaling) or myeloid disease (NPM1)^93^ (Fig. 3).

Moreover, *TET2* mutations are detected in B cells from AITL or other cancer patients, which may show a restricted repertoire of hyper-mutated IG genes^94^ and can harbor additional somatic mutations, notably in NOTCH1^36^. Whether these mutations contribute to the development of B-cell lymphoproliferative diseases frequently observed during the course of AITL disease is still under investigation.

### Therapeutic options

Despite the unique clinical and pathological characteristics of AITL, the therapeutic approaches are currently similar to those applied for all peripheral T-cell lymphomas. A recent overview of the mechanisms involved in different PTCLs and novel treatments under evaluation underlines the difficulty of targeted treatment^95^. However, due to the rareness of this lymphoma, the best data available to guide treatment are based on prospective studies. The first recommended therapy is based on chemotherapeutic agents, such as the combination of cyclophosphamide, doxorubicin, vincristine and prednisone (CHOP therapy). However, this strategy failed to increase the survival rate to more than 30% at 5 year, mainly due to high level of relapse. Several attempts to improve this strategy have included combining chemotherapeutic agents (CHOP) with other anti-cancer molecules but no further improvement in survival was obtained so far.

Different molecules targeting Tfh differentiation and function were evaluated^96,97^. In addition, several molecules have been tested for their capacities to target different cellular component of the tumor microenvironment (Anti-CD20; anti-VEGF)^98,99^. Another therapeutic option is the transplantation of autologous or allogenic hematopoietic stem cells in young patients after relapse to treatment. However, this approach knows limited success and is still controversial^100^.

After relapse, prognosis is poor for AITL patients with a median survival inferior to 6 months^101^. Some drugs have been approved by the FDA, such as pralatrexate^102^, and the histone deacetylase inhibitors romidepsin^103^ and belinostat^104^, but they resulted in limited activity. Given the high frequency of mutations targeting the DNA methylation/hydroxymethylation pathway, treatment with 5-azacytidine, a hypo-methylating agent, has been evaluated as single agent in a retrospective cohort^40^ or in combination with romidepsin^105^ giving promising results. The oral form of 5-azacytidine is currently being evaluated in an ongoing phase 3 study (NCT03593018). In myelodysplastic syndrome hypo-methylating agents are also in clinical use and they seem to be more effective in *TET2*-mutated cases^106,107^. This points toward a possibly effective treatment for *TET2-*mutated AITL patients. Indeed, several AITL patients have benefited from treatment with azacytidine, a demethylating agent^40,108,109^. However, this is not per se related to the *TET2* mutational status in AITL. Gregory et al. reported for example a durable remission upon 5-azacytidine treatment in an AITL patient that did not carry *TET2* mutations^110^. This suggests that 5-azacytidine could also show efficacy in DNMT3A/IDH2 germline patients, who can, however, harbor epigenetic changes, especially 5 hmC loss^111^.

In addition to epigenetic targeting, TCR-signaling targeting with tyrosine kinase inhibitors is under evaluation. Promising efficacy was reported for Duvelisib, a PI3K inhibitor, in relapsed/refractory patients^99^, and a phase I clinical trial was reported in 2020 using Dasatinib, a multi-kinase inhibitor of TCR signaling resulting in partial responses in these patients^98^.

Development of antibodies targeting immune checkpoint, especially PD1, has been a recent breakthrough in the cancer field, inducing prolonged remission in cancers unresponsive to conventional treatments. However, the usage of anti-PD1 therapies in AITL remains controversial. Indeed, targeting the immune system in AITL is by far more complex than in other cancers, because the neoplastic targets are the PD1 expressing T cells themselves. Furthermore, recent results suggest that PD1 could act as a potent tumor suppressor in PTCL^112^, suggesting that blocking PD1 on neoplastic T cells could result in tumor cell proliferation and lymphoma progression. Clinical trials are ongoing and should clarify the place of immune checkpoint in AITL treatment. In addition, further targeted therapies are being developed and tested in ongoing clinical trials and are reviewed by others^12,95,113–115^.

Finally, the lack of efficient treatment for AITL patients, emphasizes the need for supplemental therapeutic options. Several of the mouse models described below have been developed not only to better understand the pathogenesis of AITL, but also with the aim to test new compounds.

## Preclinical mouse models for AITL

### The Roquin^san^ mouse model

Vinuesa et al.^116^ developed in 2005 the sanroque mice strain that bears a homozygous point mutation in the *Roquin/Rc3h1 gene*. In these *Roquin*^*san/san*^ an accessive accumulation of Tfh cells was detected accompanied by systemic autoimmune disease marked by lymphadenopathy, splenomegaly and IgG increase, features resembling AITL disease. However, it was only much later that the mice heterozygous for the ‘san’ allele (*Roquin*^*san/+*^) were studied, which was reported as one of first animal models mimicking AITL^117^. Fifty-three percent of the *Roquin*^*san/+*^ mice that developed AITL-like disease were characterized by multiple features of AITL tumors such as lymphadenopathy, perturbed nodal architecture, prominent vascularization, splenomegaly, hypergammaglobulinemia, and increased numbers of oligoclonal or clonal proliferating Tfh cells (PD1^high^ CXCR5^+^ Bcl-6^+^) accompanied by GC B cells. Noteworthy, FDC arborization, a prominent feature of AITL, was absent in these *Roquin*^*san/+*^ tumors.

However, a mutation in *Roquin* was not detected in human AITL patients^118^, suggesting that mutations in other genes and pathways are involved in AITL disease establishment.

Nevertheless, this was the first preclinical model mimicking AITL disease, available for preclinical testing of new medicines.

### Swiss Jim Lambert (SJL) mouse model

Multiple studies have revealed SJL mice as a valid model for malignant lymphoproliferative tumors. In these mice the germinal center (GC) B cells express the vSAg superantigen of a mammary tumor virus (mtv-29), which is known for its capacity to stimulate CD4^+^ T cells, which will in turn induce B-cell proliferation^119,120^. Thanks to this T-B-cell activation/interactions, 90% of these mice develop a lymphoproliferative disease, similar to non-Hodgkin’s lymphomas^121^.

Jain and colleagues^122^ though were the first to describe a similarity between the SJL lymphoproliferative malignancies and the characteristics of AITL. They put forward the role of IL-21, a hallmark cytokine for Tfh development^123,124^ in this pathology. Indeed, from 12 months on, SJL mice demonstrated a marked change in splenic and lymph node architecture. Immuno-phenotyping demonstrated a clear augmentation of Tfh cells (CD4^+^CXCR5^+^ICOS^+^ PD1^+^ cells) and GC B cells in the spleens of diseased mice. The Tfh cells showed a clonal rearrangement of V segments. Transcriptomic analysis showed a clear overexpression of genes implicated in Tfh signaling such as IL-21 and IL-10 and genes coding for the chemokines CCL8 and CCL12, which attract macrophages and dendritic cells. To confirm the implication of IL-21 in this AITL-like pathology, Jain et al.^122^ generated an IL-21 receptor knock-out mice. In these mice the CD4^+^ Tfh frequency was reduced significantly associated with the disappearance of B cells. Although the SJL mouse model has only some characteristics of AITL, it highlighted the importance of IL-21 signaling in the establishment of the Tfh component of this T-cell lymphoma.

### Inactivating *Tet2*: the *Tet2*^*−/−*^ mouse model

As mentioned previously, *Tet2* mutations were identified in 80% of the AITL patients^45,47,67^. These mutations lead to the loss of Tet2 function and are thus associated with aberrant methylation of certain genes implicated in tumoral progression. Though several other groups developed *Tet2* KO mouse models, these did not spontaneously result in AITL-like disease^61,65^. Muto and colleagues^66^ generated their *Tet2*^*−/−*^ mouse model in order to describe in detail the consequences of Tet2 inactivating mutations on cancer. They aged the mice for 40–60 weeks and though no abnormalities were identified in the peripheral blood, these mice showed a major enlargement of the spleen and lymph nodes as in AITL. Flow cytometry analysis demonstrated an increase of T-cell numbers but also the presence of specific Tfh cell markers at their surface (PD1^+^CXCR5^+^) with a clonal rearrangement of the TCRβ chain. Diseased *Tet2*^−/−^ mice showed a low proportion of B cells and no increase in IgGs was detected, so these common AITL disease features were missing.

Transcriptomic analysis revealed an enrichment in genes implicated in Tfh differentiation and in genes coding for pro-inflammatory cytokines. Since the role of Tet2 as an epigenetic regulator is well known, the methylation profile was studied in the lymphomas of these mice. Interestingly, hypermethylation of the first intron of Bcl-6 was detected, which translated in overexpression of this gene. This might explain the abnormal outgrowth of Tfh cells since Bcl-6 is essential for Tfh development^21^ and it promotes the expression of CXCR5 and PD1, two Tfh cell markers^23,123^. This shows a striking similarity with AITL, in which the Bcl-6 locus is often hypermethylated^125^.

In summary, although the *Tet2*^−/−^ mice developed a T-cell lymphoma with a predominant Tfh phenotype and marked epigenetic changes, it did not recapitulate the full characteristics of the human AITL disease since GC B cells were not a major component of the lymphoma and other clinical manifestation such as increased IgGs or inflammatory cytokine secretion typical of AITL were not present in this model.

### *IDH2* R172K mutated mouse model

Mutations in isocitrate dehydrogenase *IDH1* and *IDH2* contribute to malignant progression by producing the oncometabolite D2-HG^49,82^. In AITL, *IDH* mutations are almost restricted to *IDH2*^R172K ^^45,49^. Since few studies evaluated the role of *IDH2* mutation in AITL, Lemonnier et al.^81^ generated a heterozygous Vav-cre *Idh2*^R172K^ mice expressing the mutated *Idh2* in the whole hematopoietic system. This mutation led to higher 2-HG levels in the serum and higher intracellular 2-HG levels in T, B, and myeloid cells from *Idh2*^R172K^ mice as compared with WT littermates. Moreover, this mutation impaired thymic development and in the spleen total T-cell numbers remained the same but B-cell numbers decreased. However, CD4^+^ and CD8^+^ naive T cell were decreased, while CD8^+^ central memory cells were increased. No significant changes in Tfh cell proportions nor lymphoma development was detected. Of interest though, 2-HG inhibits TET family enzymes, which in turn results in lowered production of 5-hydroxymethylcytosine (5hmC) in DNA. It is thought that these epigenetic changes lead to malignant transformation of the hematopoietic system. Indeed, Lemonnier et al.^81^ showed a strong increase of 5 hmC in the DNA of T cells from the *Idh2*^R172K^ mice compared to controls. Although this model does not recapitulate AITL features, it must be noted that the mice investigated were only between 3 and 7 months and it would be interesting to characterize *Idh2*^R172K^ mice at later time-points.

### Inactivation of *Tet2* combined with overexpression of *RhoA* G17V

As described above, multiple studies indicate that *TET2* is the most frequently mutated gene in AITL and that mutations in *RHOA* (G17V) frequently coincides with TET2 mutations in patients^44,45^. Therefore, three independent research groups have invested in mouse models carrying *Tet2* knock-out in the T cells or in the whole hematopoietic system combined with the expression of the mutant *RhoA* gene (G17V)^54,58,68^.

The first model was generated by Zang and colleagues^54^. They revealed that combining the genetic inactivation of *Tet2* associated with the expression of mutant RhoA^G17V^ in murine mature T cells resulted in aberrant T-cell development. T cells from *WT* or *Tet2*^−/−^ mice were transduced with a retroviral vector encoding for *RhoA*^G17V^ or not and transferred to recipient mice. Interestingly, adaptive transfer of these *Tet2*^−/−^ *RhoA*^G17V^ T cells into mice led to reduced life span of the animals (21 weeks). They displayed skin ulcers, enlarged lymph nodes, infiltration of both T and B cells in different organs. Strikingly, an increase in GC B cells and the inflammatory cytokine IL-6 were observed. This recapitulates some of the immune phenotypes detected in AITL patients.

Moreover, strongly proliferating Tfh cells were identified in the peripheral lymphoid tissues, at cost of other CD4^+^ T-cell types, a typical feature of human AITL. No marked effect on CD8^+^ T cells was seen. Moreover, *Tet2*^*−/−*^ *RhoA*^*G17V*^ T cells from tumors partially recapitulated the expression signature of human AITL tumor cells (Table 1).

**Table 1:** Preclinical mouse models of AITL disease.

Transgenic mice	*Roquin*^*san/+* ^^116^	*SJL*^122^	*Tet2*^*−/−* ^^66^	*IDH2*^*R172K* ^^81^	*RhoA*^*G17V*^ *Tet2*^*−/−*^			Plck-GAPDH^135^
					*RhoA*^*G17V*^*;* *Tet2*^*−/−*^ T-cell transfer^54^	*Tet2*^*−/−*^ *BM* + *RhoA*^*G17V*^ transfer^58^	*CD4-RhoA*^*G17V*^*;Tet2*^*−/−*^^ 68^	
Clinical features: lymphoma development	>4 months T-cell lymphoma non-malignant	>11 months DLBCL Cytomas/sarcomas	>60 weeks T cell lymphoma	3–7 months No malignant T cell lymphoma	<21 weeks No malignant T cell lymphoma	<12 months Malignant T-cell lymphoma	>12 months Malignant T cell lymphoma Tail fibrosis	>18 months Malignant T cell lymphoma Skin rash Abdominal ascites
Pathological features	Splenomegaly Lympho-adenopathy Disorganized architecture of spleen and LN Vascularization LN Oligoclonal or clonal T cells	SplenomegalyLympho -adenopathy Disorganized architecture of spleen and LN T cell and B-cell clonality (DLBCL)	Enlarged spleen + LNs Nodules in liver Destroyed follicular structures T cell clonality	Effect on T cell differentiation: increase CD8_cm_ and decrease CD4_n_ and CD8_n_	Skin ulcers Enlarged LN Increase of germinal center in spleen and lymph nodes Infiltration T, B in other organs	Splenomegaly Enlarged LNAltered architecture of spleen and LN FDC network Vascularization LN T cell clonality Inflammatory cytokines: IL-6, TNFα	Splenomegaly Lympho adenopathy Liver infiltration Vascularization LN Autoimmune feature: autoantibodies (dsDNA increase in blood) T cell clonality	Splenomegaly Hepatomegaly Liver infiltration Enlarged LN + ectopic LN Altered architecture of spleen, liver and LN Autoimmune feature: inflammatory cytokine production (INFγ, IL-10, IL-6 + autoantibodies) T cell clonality BM infiltration
Immuno-phenotype	Tfh (CD4 + CXCR5 + PD1+, Bcl-6+) GC B (FAS+ GL-7+) Ki67+ Tfh IgG levels+ No FDC network	Tfh (CD4 + CXCR5 + ICOS + PD1+, IL-21+++) GC B cells (FAS + GL-7+) cytokines/chemokines IL-10++, CCL12, CCL8 IgG levels++	Tfh (CD4 + CXCR5 + PD1+, ICOS + Bcl-6++) Tfh gene signature B220 B cells decrease	No Tfh B220 B-cell decrease	Ki67 + CD4 T Increase CD4 + CD44 + T Tfh (CD4 + CXCR5 + Bcl-6+) Reduction T reg GC B (FAS + GL-7+) Partial AITL gene signature Cytokines: IL-6 inFγ	Tfh (CD4 + CXCR5 + PD1+, ICOS+, Bcl-6+) Tfh gene signature AITL gene signature	Tfh (CD4 + CXCR5 + PD1+, Bcl-6+)	Tfh (CD4 + CXCR5 + PD1+, ICOS+, CXCL-13+, Bcl-6+) Tfh gene signature Ki67 +proliferating T Activated memory CD4 GC B (FAS + GL-7+) GC B-cell signature Increased PC B cells AITL gene signature Hyper Immunoglobulinemia FDC (CD35+) Cytokine/chemokine: CCL5, CCL2, CCL8, CXCL-13

**Fig. 4:**
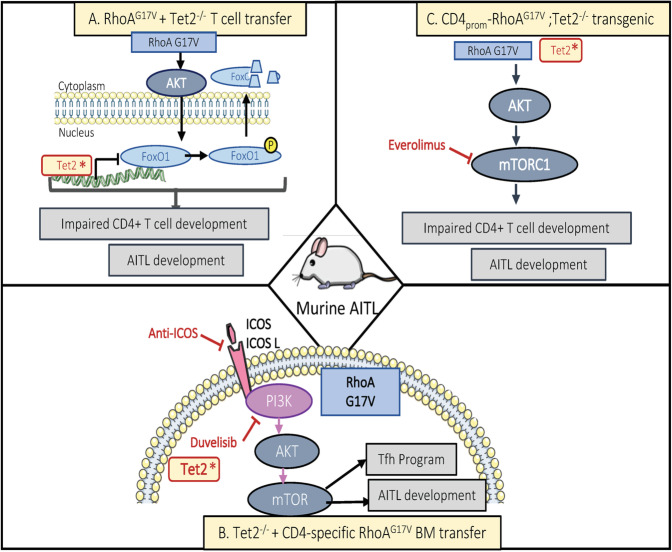
Overview of the three mouse models, mimicking the genetic inactivation of Tet2 and expression of the mutated RhoA (G17V) in AITL patients. A schematic representation of the signaling pathways upregulated in the Tfh lymphoma cells is shown. **a** AITL-like mouse model based on adaptive transfer of Tet2 invalidated mature CD4 T cells transduced with a retroviral vector expression RhoA^G17V^ (ref. ^54^). **b** AITL-like mouse model based on transplantation of the BM of *Tet2* KO mice, transduced with a retroviral vector coding for mutated *RhoA*^G17V^ (ref. ^68^). **c** AITL-like mouse model based on transgenic mice, in which Tet2 is invalidated in the whole hematopoietic system while RhoA^G17V^ under the control of a CD4-specific promoter is exclusively expressed in CD4 T cells^58^. The therapeutic agents (Everiolimus and Duvelisib) interfering with the PI3K-AKT-mTORc1 pathway are indicated.

These authors explained that the inactivation of the epigenetic modifier Tet2 associated with the expression of RhoA^G17V^ affected the transcriptional and post-transcriptional modifications of genes implicated in T cell differentiation. Comparison of expression profiles between *Tet*^*−/−*^ *RhoA*^*G17V*^ T cells and WT T cells revealed downregulation of genes associated with FoxO, a family of factors involved in T cell function regulation^126,127^. Indeed, a double inactivation of FoxO1 by hypermethylation of its promoter region (decreased transcription) and augmentation of its phosphorylated form (inducing its degradation^128^) was revealed. This suggests that *RhoA*^*G17V*^ alters Akt and subsequently increases FoxO1 phosphoryation^46,129^, while the Tet2 inactivation has an effect on its promoter activity^54^ (Fig. 4a). These genetic alterations lead to an acute inflammatory phenotype in vivo and sudden death of the animals precluding the detection of malignant transformation. Nevertheless, these data suggest a possible cooperation between epigenetic factors such as TET2 and GTPases in adult T cells which might account for inflammatory responses, typically reported for AITL patients.

To analyze the long term effect of these mutant *TET2* and *RHOA* genes in vivo, two other consecutive studies generated new mouse models allowing inducible expression of these mutations in CD4^+^ T lymphocytes or in hematopoietic progenitor cells^58,68^.

Cortes et al.^58^ engineered a knock-in mouse model for *RhoA*^G17V^ into the endogenous *RhoA* locus. To induce the expression of RhoA^G17V+^ in CD4^+^ cells, these mice were crossed with CD4CreER mice^130^. Tamoxifen induced activation of Cre recombinase results in RhoA^G17V^ expression specifically in CD4^+^ T cells. These mice showed an increase in Tfh cells identified by surface staining for CXCR5^+^ PD1^+^ Bcl-6^+^ and ICOS^+^ and expression of a more complete Tfh gene signature compared with their counterpart littermates without Tamoxifen induction. However, sole overexpressing in this model of RhoA^G17V^ was not able to recapitulate a malignant transformation of the Tfh cells. To introduce the missing factor (inactivated *Tet2*), these authors transplanted mice with the BM of *Tet2* KO mice^67^, transduced with a retroviral vector coding for mutated *RhoA*^G17V^. These mice developed evident lymphomas, enlarged spleens and lymph nodes with disturbed architecture, expanded follicular dendritic cell meshworks, increased venule arborization, all features defining AITL (Table 1). Spleen and lymph nodes demonstrated a clear presence of high number of Tfh cells and moreover monoclonal T cell populations were found in all tumor-bearing mice. These *Tet2*^−/−^ *RhoA*^G17V^ expressing tumor cells showed a clear enrichment in Tfh signature and what’s more increased expression of a more extensive signature of genes associated with human AITL. The malignant character of these cells was confirmed by transfer in secondary recipient mice, which recapitulated all the characteristics of the original AITL-like T cell lymphoma.

Ng et al.^68^ generated a second transgenic mouse model that expressed RhoA^G17V^ under the control of murine CD4 regulatory elements (CD4-*RhoA*^*G17V*^). These mice developed dermatitis and fibrosis at the ears, tails and later on at the skin. This autoimmune phenotype was due to CD4^+^ Th17 cell infiltration. In addition, the CD4-*RhoA*^G17V^ mice showed a strong increase in Tfh (CD4^+^CXCR5^+^PD1^+^ICOS^+^) and regulatory T follicular cells (CD4^+^CXCR5^+^PD1^+^Foxp3^+^) compared to WT littermates at 10 weeks of age and these numbers increased with age. This already suggest a dominant role of the RhoA^G17V^ in the establishment of Tfh AITL lymphoma cells. In addition, CD4 restrictive RhoA^G17V^ expression resulted in humoral autoimmunity in older mice, a prominent feature in human AITL.

To mimic concurrent mutation of *Tet2* and *RhoA*, they adapted a similar approach to Cortes et al.^58^. CD4-*RhoA*^G17V^ mice were crossed with *Tet2*^fl/fl^; Vav-Cre^+^ mice^65^, which harbor a deleted *tet2* throughout the whole hematopoietic system. This model is thought to recapitulate the sequence of events seen in AITL patients, in which *Tet2* was invalidated in all HSCs and lineages derived thereof, while RhoA^G17V^ was only detected in the mature T cells^45,131^. These CD4-*RhoA*^G17V^*Tet2*^−/−^ mice model recapitulated the same characteristics of the CD4-*RhoA*^G17V^ model mentioned above. To mimic chronic T cell stimulation as seen in AITL, CD4-*RhoA*^G17V^*Tet2*^−/−^ mice were crossed with OT-II mice^132^ and monthly immunized with ovalbumin, upon which they developed T cell lymphomas (Table 1). Diseased mice were marked by lymphoadenopathy, splenomegaly, enlarged lymph nodes, infiltration of the liver by CD4^+^ T cells. And more characteristic features of AITL were present: formation of venules, pronounced augmentation of Tfh cells, with clonal TCRγ rarrangement. Ng et al.^68^ concluded thus that RhoA^G17V^ in CD4 T cells in cooperation with *Tet2* loss-of-function mutations induces the development of Tfh-like lymphomas very similar to human AITL.

Remarkably, both Ng et al.^68^ and Cortes et al.^58^ revealed the activation of the same signaling pathway in their respective AITL preclinical models. In these two independently generated models they detected increased mTOR activity in the *Tet2*^−/−^ *RhoA*^G17V^ Tfh tumor cells, which underlined the equivalence of these genetic preclinical AITL models.

Cortes et al.^58^ showed even that the appearance of Tfh was correlated with an increased ICOS expression in *Tet2*^−/−^ *RhoA*^G17V^ expressing tumor cells. They revealed a role for the *RhoA*^G17V^ mutation in the induction of ICOS, which in turn regulates PI3K-mTOR signaling (Fig. 3b). Interestingly, in the *Roquin*^*san*^ AITL-like mouse model (see the “The Roquin^san^ mouse model” section), it was reported that mutant Roquin^san^ binds ICOS mRNA with higher affinity than its wt counterpart, protecting it from mRNA degradation^117^. Thus, this study also confirmed that high ICOS expression on CD4 resulted in accumulation of Tfh cells.

Therefore, Cortes et al.^58^ proposed that this PI3K-mTOR signaling pathway played a role in AITL pathogenesis. They evaluated a new therapeutic option, based on inhibition of this pathway by the drug Duvelisib (PI3K inhibitor), which blocked tumor development in vivo in *Tet2*^−/−^ *RhoA*^G17V^ lymphoma bearing mice. Ng et al.^68^ and colleagues tested the activity of the mTOR inhibitor Everolimus in mice transplanted with the CD4-*RhoA*^G17V^*Tet2*^−/−^; OT-II tumors with a strong reduction of tumor burden as a result and increased survival of the mice (Fig. 4c). The study of Zang et al.^54^ showed an altered expression/function of FoxO1 (Fig. 4a). This is in agreement with increased mTOR activity in AITL-like tumor Tfh cells found in the two above studies since FoxO1 is a negative regulator of mTORc1. Although these three studies indicate a relationship between mTORc1 activity in Tfh differentiation and/or maintenance this question is still not resolved. In addition, another independent research team confirmed more recently the role of FOXO1 in tumorigenesis and proliferation of AITL^133^.

In summary, two independent studies generated AITL preclinical mouse models indicating that targeting the PI3K-mTOR pathway at different levels may be a new therapeutic option for AITL patients. In line with these results, a clinical trial including 3 AITL patients showed that 2 out of 3 responded to Duvelisib^96^. A new trial is ongoing that will include RhoA genotyping to confirm a link with the RhoA^G17V^ induced PI3K-mTOR in AITL patients (NCT02783625). Using these genetic AITL mouse model, Nguyen et al.^134^ found that Dasatinib, a multi-kinase inhibitor prolonged their survival through inhibition of hyperactivated TCR signaling. A phase I clinical trial study of dasatinib monotherapy was therefore engaged and showed responses in five relapsed/refractory AITL patients.

### GAPDH overexpression in T-cell lineage: plck-GAPDH mice

Mondragon et al.^135^ had a completely different approach and generated a new mouse model for AITL to some extent by chance. To study the role of metabolism in T cell differentiation and malignancy, these authors decided to generate a mouse that overexpressed a key enzyme of glycolysis, glyceraldehyde 3-phosphate dehydrogenase (GAPDH), controlled by the T cell specific promoter, plck (plck-GAPDH mice)^135^. GAPDH a central enzyme of the glycolytic pathway has indeed remerged as a key factor in T cell survival, activation and function^136–139^.

Though no major perturbation in very early T cell development and up to 1 year was detected, mice reaching 2 years of age, developed suddenly lymphomas accompanied by skin rash and ascites accumulating in the abdomen. These mice developed indeed splenomegaly, hepatomegaly and enlarged lymph nodes, showing an altered architecture with the follicular structure destroyed by infiltration of T and B cells with increased CD4^+^ and dendritic cell numbers. The combination of all these clinico-pathological symptoms suggested that the plck-GAPDH mice developed an AITL-like peripheral T cell lymphoma (PTCL).

Resemblance with human AITL disease increased upon immune-phenotyping showing that the tumor mouse tissues (spleen, liver, lymph nodes) were marked by a specific increase of CD4^+^PD1^+^CXCR5^+^ICOS^+^CXCL-13^+^Bcl-6^+^ T follicular helper (Tfh) cells as compared to WT. TCRβ clonality was confirmed in the CD4^+^PD1^+^CXCR5^+^ T cells for different plck-GAPDH tumors confirming a true T cell lymphoma. B-cell phenotyping in the enlarged spleens and lymph nodes of the plck-GAPDH mice demonstrated dominance of hyperplasic FAS^+^ GL-7^+^ GC B cells and a higher number of plasma B cells compared to WT. Gene expression profiling confirmed a clear upregulation of Tfh and GC signature genes in the tumors. This coincided with presence of increased IgGs, and autoantibodies in the serum of diseased mice. In accordance, increased expression levels of inflammatory and anti-inflammatory cytokines were comparable with a human AITL enriched inflammation signature.

Since resemblance with AITL was striking, these authors analyzed the Tfh plck-GAPDH lymphoma cells for mutations in *RhoA, Tet2, Idh2*, and *Dmt3a*, frequently mutated in these patients^79,140^. Though the AITL hotspot mutation *RhoA*^G17V^ was not present, two mutations in *RhoA* were found, 1 point mutation at position 37 (*RhoA*^T37M^) present in all tumors and an INDEL frameshift mutation in 4/15 lymphomas. *RhoA*^T37M^ was as *RhoA*^G17V^ inactivated for binding of GTP. Whole exome sequencing did not reveal further mutations in the epigenetic modifiers *Tet2, Idh2*, and *Dmt3a*.

As evidenced by Cortes et al.^58^ and Ng et al.^68^, a mutation in *RhoA* invalidating its binding to GTP such as shown for *RhoA*^T37M^ or *RhoA*^G17V^ once again is correlated with the uncontrolled outgrow of Tfh lymphoma cells.

**Fig. 5:**
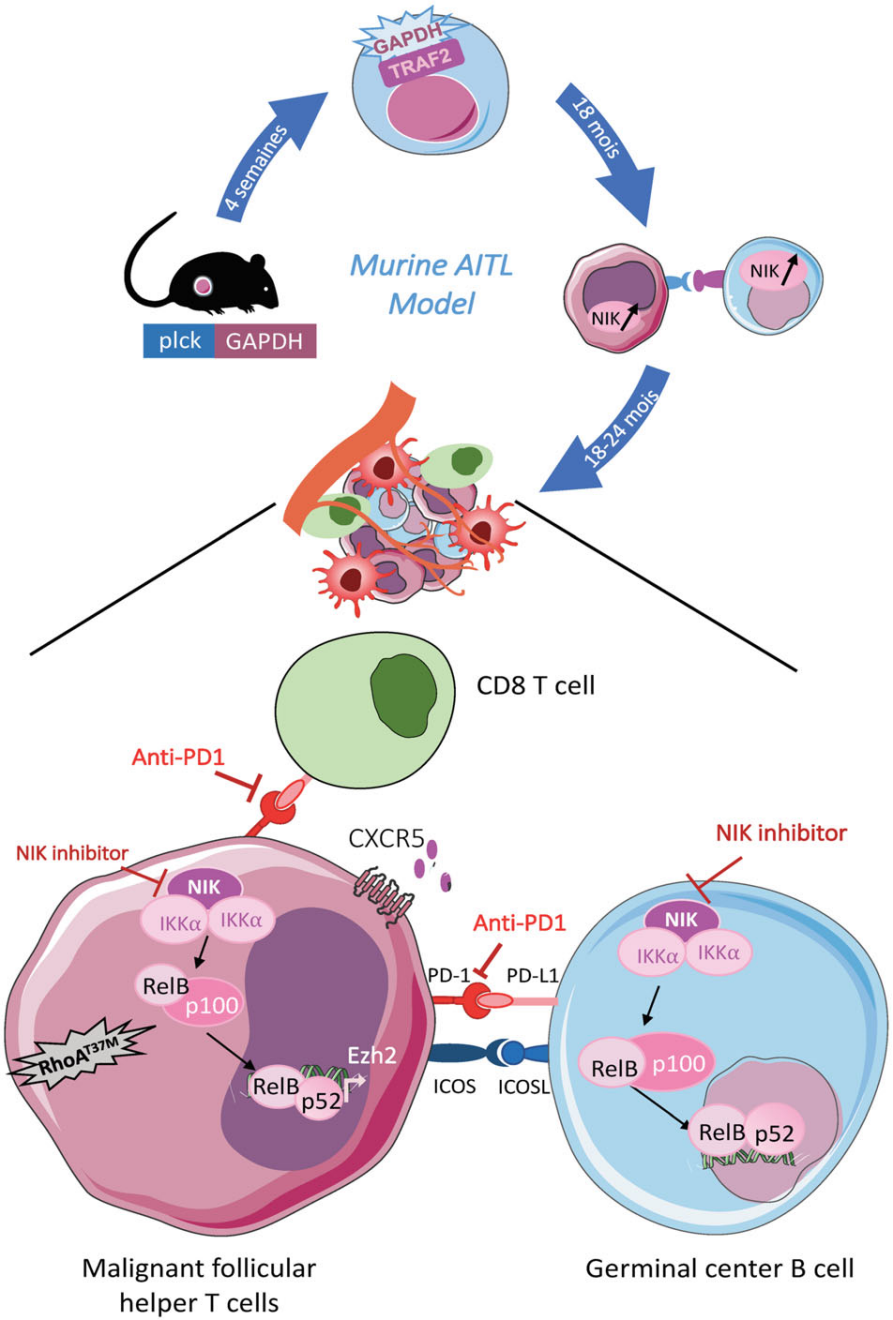
AITL-like mouse model generated by overexpression of the glycolytic enzyme GAPDH in the T cell lineage. Initially, through binding of GAPDH to TRAF2 the canonical NF-κB pathway is upregulated, which persist for 18–24 months, inducing an inflammatory environment. This continuous upregulation induced a strong upregulation of the noncanonical pathway revealed by NIK upregulation in the Tfh lymphoma cells accompanied by germinal center B cells, which also show NIK upregulation. A schematic representation of the signaling pathway upregulated in the Tfh lymphoma cells is shown. A therapeutic agent (NIK inhibitor) interfering with the noncanonical NF-kB pathway is indicated as also the treatment with a checkpoint inhibitor anti-PD1.

The remaining question was though how did GAPDH overexpression in T cells lead to this AITL lymphoma? The inflammatory cytokine profile in the plck-GAPDH tumors pointed toward activation of the NF-κB pathway. Indeed, in young mice it was show that GAPDH could associate with NF-κB canonical pathway member TRAF2 and induce its continued activation in these mice (Fig. 5). However, in the plck-GAPDH lymphoma a dominant upregulation of the noncanonical NF-κB pathway was detected. These authors therefore hypothesized that AITL disease was established in plck-GAPDH due to a chronic activation of the canonical NF-κB pathway and that in turn this leads to upregulation of its noncanonical counterpart. They reinforced this hypothesis by demonstrating that the genetic chronical activation of the NF-κB canonical pathway in aged plck-*IκB*^−/−^ mice led to noncanonical pathway activation in Tfh cells. In addition, the chronic activation of the NF-κB pathway led to an inflammatory environment possibly prosperous to induction of genetic mutations such as the one detected in the GPTase, RhoA.

Strikingly, these authors demonstrated that the noncanonical NF-κB pathway is highly upregulated in human AITL, equivalent to their murine AITL model. In agreement with these results, Valois et al.^51^ showed that *CARD11* mutations seem to be at the origin of constitutive NF-κB signaling and might assist in tumor outgrow in AITL.

Therefore, therapeutic intervention with this pathway was evaluated. Activation of the noncanonical pathway depends on the activation of the NF-κB inducing kinase (NIK) and IKKα phosphorylation^141^. Mondragon et al.^135^ opted for a newly developed small-molecule inhibitor of NIK to interfere with this pathway (patent PCT/EP2017/067306; Fig. 5). The plck-GAPDH AITL mice showed increased survival and lower tumor burden upon NIK inhibition. However, to wipe out completely the Tfh lymphoma CD4^+^ PD1^+^ cells a combination of NIK inhibitor and the checkpoint inhibitor anti-PD1 was required^142,143^. The latter reactivated cytotoxic CD8 T cells in the tumor. As mentioned above, anti-PD1 therapies in PTCL/AITL remains controversial since recent results suggest that PD1 could act as a potent tumor suppressor in PTCL^112^, suggesting that anti-PD1 treatment could result in lymphoma progression. To clarify if checkpoint inhibition is efficient and safe in PTCL, ongoing clinical trails will bring clarity in the future. The validity of this AITL model and the relevance of NIK inhibition for this disease was confirmed by a clear effect of the NIK inhibitor on the reduced survival of CD4^+^ Tfh cells and B cells in human AITL biopsies ex vivo. This provides a clear rationale for use of specific NF-κB inhibitors in AITL. A positive outcome in using this agent might be due to the fact that this inhibitor is not only targeting T cell but also the GC B cells (also marked by high levels of NIK^144^) in the tumor microenvironment. Currently, the novel NIK inhibitor is not available yet for clinical application. However, efforts to produce a clinically compatible NIK inhibitor are ongoing and not only for treatment of rare lymphomas such as AITL, but also for melanomas and other cancers, which are strongly addicted to the noncanonical NF-κB pathway^145^.

### AITL patient-derived-xenograft (PDX) mice

AITL patient-derived-xenograft (PDX) mice might represent a highly valuable patient lymphoma-based model for drug testing. Sato et al.^146^ and Townsend et al.^147^ were able to obtain primary engraftment of human AITL lymphomas into immunodeficient NOD/SCID γC^−/−^ (NSG) mice. These primary xenografts consisted of human Tfh PD1^+^ CD4^+^ cells and cells from the TME (B cells, CD8 T cells, plasmocytes secreting IgGs), very similar to the engrafted original human AITL tumor. Unfortunately, in subsequent secondary and tertiary engraftments into NSG recipient mice, the TME cells gradually got lost or were strongly reduced in numbers, which questions to some extend the reliability of these PDX mice for testing of novel drugs such as immunotherapies in the absence of the tumor accompanying cells. Especially, since the TME cells sustain survival and proliferation of the tumoral Tfh cells as also the immune surveillance of the tumor. Continuous efforts are made to improve these models. It need to be mentioned that AITL cells capable of engraftment into NSG mice are made available in a public depository of xenografts, which are precisely annotated in terms of genomic and immunophenotypic features (www.proxe.org)^147^.

However, for now, the current AITL PDX mouse models lacking TME cells do not seem appropriate for testing of some medicinal products such as immune checkpoint inhibitors.

## Conclusion

Because of its complexity due to the implication of multiple cell types in the TME, evaluation of new therapeutic options for AITL in vitro are not reliable.

Only very recently, in 2018 and 2019, three valid preclinical mouse models for AITL were engineered. Two mouse models^58,68^ are based on genetic inactivation of Tet2 combined with overexpression of the mutated RhoA^G17V^ and one model relies on overexpression of GAPDH in the T cell lineage^135^. These lymphoma mouse models closely mimicked AITL disease in terms of clinical, pathological, histological, transcriptional, genetic, and immunophenotypic features. Moreover, these models allowed to reveal that AITL tumor were addicted to activation of signaling pathways such as mTOR^58,68^ or NF-κB^135^ and permitted to test successfully therapeutic options interfering with these pathways. This means that finally we can make use of these AITL preclinical mouse models to address questions otherwise still out of reach. These new AITL mouse models will provide the missing link between the proof of concept of complex novel therapies and their translation into the clinic.
